# Hydrogen Sulfide Protects against Chemical Hypoxia-Induced Injury via Attenuation of ROS-Mediated Ca^2+^ Overload and Mitochondrial Dysfunction in Human Bronchial Epithelial Cells

**DOI:** 10.1155/2018/2070971

**Published:** 2018-09-30

**Authors:** Cai-Xia Liu, Yu-Rong Tan, Yang Xiang, Chi Liu, Xiao-Ai Liu, Xiao-Qun Qin

**Affiliations:** ^1^Department of Physiology, Xiangya School of Medicine, Central South University, Changsha, China; ^2^Department of Microbiology, Xiangya School of Medicine, Central South University, Changsha, China; ^3^Institute of Nursing, Guangdong Food and Drug Vocational College, Guangzhou 510000, China

## Abstract

Oxidative stress induced by hypoxia/ischemia resulted in the excessive reactive oxygen species (ROS) and the relative inadequate antioxidants. As the initial barrier to environmental pollutants and allergic stimuli, airway epithelial cell is vulnerable to oxidative stress. In recent years, the antioxidant effect of hydrogen sulfide (H_2_S) has attracted much attention. Therefore, in this study, we explored the impact of H_2_S on CoCl_2_-induced cell injury in 16HBE14o- cells. The effect of CoCl_2_ on the cell viability was detected by Cell Counting Kit (CCK-8) and the level of ROS in 16HBE14o- cells in response to varying doses (100–1000* μ*mol/L) of CoCl_2_ (a common chemical mimic of hypoxia) was measured by using fluorescent probe DCFH-DA. It was shown that, in 16HBE14o- cells, CoCl_2_ acutely increased the ROS content in a dose-dependent manner, and the increased ROS was inhibited by the NaHS (as a donor of H_2_S). Moreover, the calcium ion fluorescence probe Fura-2/AM and fluorescence dye Rh123 were used to investigate the intracellular calcium concentration ([Ca^2+^]_i_) and mitochondria membrane potential (MMP) in 16HBE14o- cells, respectively. In addition, we examined apoptosis of 16HBE14o- cells with Hoechst 33342. The results showed that the CoCl_2_ effectively elevated the Ca^2+^ influx, declined the MMP, and aggravated apoptosis, which were abrogated by NaHS. These results demonstrate that H_2_S could attenuate CoCl_2_-induced hypoxia injury via reducing ROS to perform an agonistic role for the Ca^2+^ influx and MMP dissipation.

## 1. Introduction

Hypoxia or ischemia causes an imbalance between reactive oxygen species (ROS) and the antioxidant defense system leading to oxidative stress, which is closely linked to the pathogenesis of acute and chronic airway disorders [1]. Exposure to oxidants may cause a free radical-initiated lipid peroxidation and induce rapid changes in membrane lipid composition in the serum, heart, lung, liver, and kidney of hypoxic rats [2–4]. Increased amount of ROS has been directly linked to oxidation of proteins, DNA, and lipids, which may induce inflammation and direct cellular injury through regulation of multiple proinflammatory and anti-inflammatory mediators in the various tissues especially in the airway and lung. Growing evidence suggests that accumulative ROS [5–8] and proinflammatory mediators may increase the airway microvascular permeability and mucus secretion and lead to the development of airway hypersensitivity and the remodeling of extracellular matrix and blood vessels [9]. Intracellular excessive ROS induced by hypoxia leads to intracellular Ca^2+^ accumulation [10] and a decrease in mitochondrial membrane potential (MMP) via promoting the open of mitochondrial membrane permeability transition pore [11, 12] and results in cellular injury, reduced cell survival, and induced cell apoptosis.

Hydrogen sulfide (H_2_S) is a flammable, malodorous gas with the smell of rotten eggs and initially classified as an environmental hazard. However, H_2_S has been identified as the third endogenous signaling gasotransmitter (after NO and CO) produced by a series of enzymes in mammals and has efficiently anti-oxidative, anti-inflammatory properties [13]. Accumulating evidence shows that H_2_S presents a protective effect against oxidative stress on various tissues such as kidney [14], central nervous system [15–17], cardiomyocytes [18, 19], lung [20, 21], and so on. Moreover, H_2_S increases the level of glutathione (GSH) by enhancing the activity of *γ*-glutamyl-cysteine synthetase [22] and scavenges superoxide free radical, hydrogen peroxide (H_2_O_2_) and reduces the accumulation of lipid peroxidations [23].

The airway epithelial cell is the initial cell type encountered by inhaled environmental factors and medications for the treatment of airway diseases. The deciduous ciliated cells in asthma patients suggest that the patients' airway epithelial barrier is often compromised [24, 25]. As the first physical barrier, the airway epithelia preventing invasion of inhaled environmental agents are easily stressed, damaged, and even denuded. An inability to recover intercellular contacts and deficient repair response after injury may be responsible for the activated and damaged phenotype of the asthma bronchial epithelium. Nevertheless, the effects of H_2_S in the airway against oxidative stress remain unclear. Our study focused on the effects of H_2_S by using the sodium hydrosulfide (NaHS) as a donor on cell injury induced by Cobalt Chloride (CoCl_2_), a commonly used chemical reagent in establishing hypoxia models* in vitro*, in human bronchial epithelial cell line (16HBE14o-).

## 2. Materials and Methods

### 2.1. Cells Culture

The immortalized human bronchial epithelial cell line 16HBE14o- was purchased from the cell bank in central laboratory of Central South University. Cells were maintained in a mixture medium of DMEM:F12 (1:1) (Hyclone, USA) with 3.15 mg/L glucose and supplemented with 100 U/ml penicillin, 100 U/ml streptomycin, and 10% fetal bovine serum (Hyclone, USA) and incubated at 37°C in a humidified 5% CO_2_ atmosphere. 16HBE14o- cells during a logarithmic growth phase were treated with NaHS (Sigma-Aldrich, USA) and N-acetyl-L-cysteine (NAC, ROS scavenger, Beyotime Institute of Biotechnology, China) for 30 min or 60 min prior to exposure to CoCl_2_ (Sigma-Aldrich, USA), respectively.

### 2.2. Detection of Cell Viability by CCK-8 Assay

The effect of CoCl_2_ on the cell viability was measured by the Cell Counting Kit (CCK-8) [26] and cells were cultured in the same condition as above. Cells were treated with or without CoCl_2_ in the presence or absence of NaHS. Briefly, cells were counted, adjusted, and seeded in a 96-well plate. Cells were seeded in five copies for each treatment group. The cells in control group were treated with an equal volume (100* μ*l) of DMEM. 10* μ*l CCK-8 solution was added to each well according to the manufacturer's protocol and then the cell culture plate was incubated for 1–4 h. The optical density at 450 nm was detected with a microplate reader (Thermo Fisher Scientific, USA). Each determination was performed at least three times.

### 2.3. Measurement of Intracellular ROS Content

Intracellular ROS levels were measured in reference to previous reports [15]. After the incubation with 5* μ*mol/L DCFH-DA for 50 min at 37°C in dark, cells in a logarithmic growth phase (2 × 10^5^ cells per well in a confocal dish) were washed with EBSS (in mg/ L: 6800 NaCl, 400 KCl, 264 CaCl_2_·2H_2_O, 200 MgCl_2_·7H_2_O, 2200 NaHCO_3_, 140 NaH_2_PO4·H_2_O, and 1000 glucose, pH 7.2) for three times. Next, the fluorescence intensity of DCF was monitored by laser scanning confocal microscope (SP5, Leica) system and analyzed with LAS AF 2.5.1.6757 software (Germany).

### 2.4. Measurements of [Ca^2+^]_i_

[Ca^2+^]_i_ measurement in 16HBE14o- cells was performed according to previous descriptions [15]. Briefly, 16HBE14o- cells were incubated with Fura-2/AM (1* μ*M, Invitrogen, USA) at 37°C for 40 min in dark after cells inoculated in a confocal dish were treated. Then, the cells were washed with EBSS (containing 1000 mg/L glucose) for three times and examined by fluorescence inversion microscope (IX71, Olympus). Calcium ion concentration was analyzed by fluorescence microscopy imaging system (CellR-MT20, Germany) and manifested in the ratio of F340/380.

### 2.5. Measurements of MMP

MMP in 16HBE14o- cells was detected in reference to previous report [18]. In brief, Rhodamine 123 (Rh123, Beyotime Institute of Biotechnology, China) is a fluorescent dye that can be absorbed by living cellular mitochondria. The absorption values alter with the change of MMP and the fluorescence intensity of the cells. Therefore, MMP can be measured through detecting the changes of Rh123 fluorescence intensity. After the treatment, 16HBE14o- cells were incubated with 5* μ*g/ml Rh123 at 37°C for 45 min. Then, the cells were washed with EBSS (containing 1000 mg/L glucose) for three times. Three randomly selected fields were taken with a laser scanning confocal microscope (Leica, Germany). The monochromators on the excitation side were set at 488 nm and on the emission side at 525 nm. The fluorescence intensity was analyzed with LAS AF 2.5.1.6757 software (Germany). Three independent experiments were repeated.

### 2.6. Measurements of Cell Injury

Quantification of cell injury refers to the prior literature [19]. Cells were incubated with 10* μ*g/ml Hoechst 33342 (Beyotime Institute of Biotechnology, China) for 5 min after the treatment and fixed with 4% paraformaldehyde for 15 min at room temperature followed by washing with PBS twice. Uniformly stained nuclei were scored as healthy and viable cells. Condensed or fragmented nuclei were scored as damaged cells. 16HBE14o- cells were imaged with fluorescence microscope (Ti-u, Nikon). The assay was repeated for three times.

### 2.7. Statistical Analysis

All experiments were repeated at least three times. Statistical analysis was performed using SPSS 22.0. The data were presented as mean ± SD and analyzed using one-way ANOVA for multiple comparisons or* t*-test between two groups. The level of significance was set at* p* < 0.05.

## 3. Results

### 3.1. H_2_S Promoted Cell Viability of 16HBE14o- Cells Inhibited by CoCl_2_

**Figure 1:**
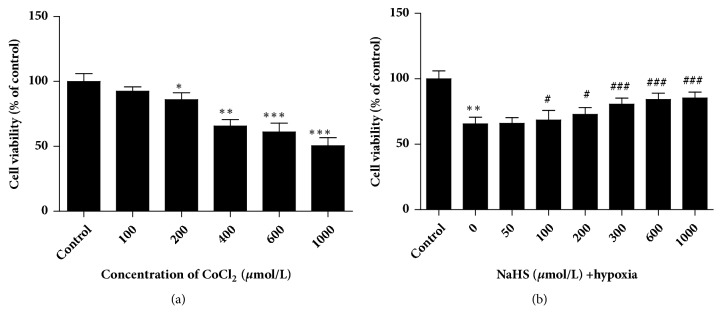
The effects of CoCl_2_ and H_2_S on the viability of 16HBE14o- cells were determined by CCK-8. (a) The viability of 16HBE14o- cells preconditioned with a series of CoCl_2_ concentration (0, 100, 200, 400, 600, or 1000* μ*mol/L). (b) The viability of 16HBE14o- cells treated with 400* μ*mol/L CoCl_2_ and NaHS in varying doses (0, 100, 200, 400, 600, or 1000* μ*mol/L) (mean± SD, n=4, *∗p* < 0.05, *∗∗p* < 0.01, *∗∗∗p* < 0.001 versus control group, #*p* < 0.05, ###*p* < 0.001 versus hypoxia group).

The results in Figure 1(a) showed that CoCl_2_-precondition had the significant dose-dependent inhibitory effect on cell viability in 16HBE14o- cells. Administration of NaHS displayed the protective effect of H_2_S on CoCl_2_-induced 16HBE14o- cell injury (Figure 1(b)). Referring to our results and related studies, we selected CoCl_2_ at the concentration of 400* μ*M [27, 28] and NaHS at the concentration of 300* μ*M [29, 30] to treat 16HBE14o-cells in the subsequent experiments.

### 3.2. H_2_S Inhibits Hypoxia-Induced ROS in 16HBE14o- Cells

**Figure 2:**
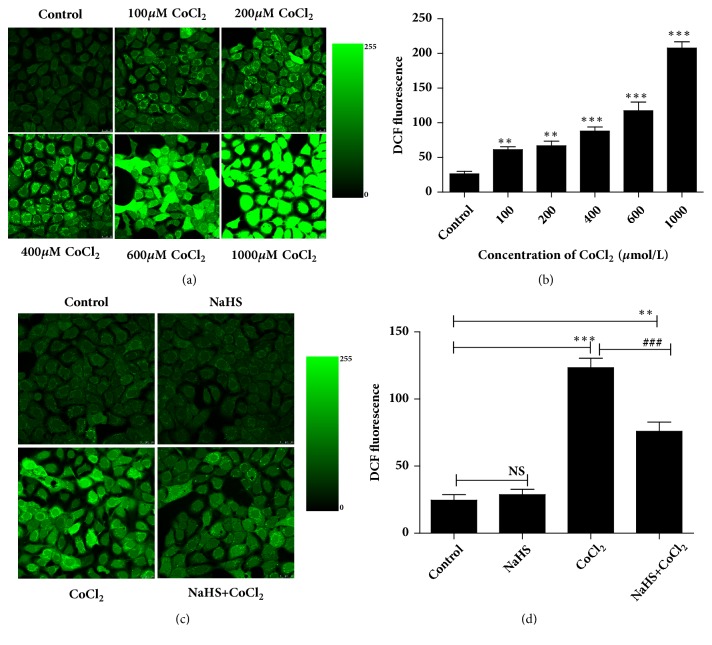
Hydrogen sulfide decreased the level of ROS induced by hypoxia in 16HBE14o- cells. Generation of ROS was determined by laser scanning confocal microscope analysis after staining the cells with DCFH-DA. (a, b) 16HBE14o- cells were pretreated with CoCl_2_ in varying doses (0, 100, 200, 400, 600, or 1000* μ*mol/L). (c, d) 16HBE14o- cells were pretreated with CoCl_2_ (400* μ*mol/L) and NaHS (300* μ*mol/L) separately or together. Results are mean values ± SD of independent experiments performed in triplicate (NS, *∗∗p* < 0.01, *∗∗∗p* < 0.001 versus control group, ###*p* < 0.001 versus hypoxia group, n=3).

DCF immunofluorescence intensity was analyzed to verify the potential role of H_2_S in hypoxia-induced intracellular ROS content. The DCF fluorescence intensity increased to 2.32-, 2.53-, 3.34-, 4.45-, and 7.88-fold of control group to correspond with the CoCl_2_ concentration of 100, 200, 400, 600, and 1000* μ*M (*p *< 0.01,* p *<0.001, Figures 2(a) and 2(b)). This demonstrated that the level of ROS was significantly elevated with a dose-dependent manner in cultured 16HBE14o- cells by pretreatment of CoCl_2_.

Next, we treated 16HBE14o- cells individually or simultaneously with 300* μ*M of NaHS and 400* μ*M of CoCl_2_ and detected the content of ROS in epithelial cells. Figures 2(c) and 2(d) showed that H_2_S had no significant effect alone but could effectively decrease the generation of ROS by 47.9% (*p *<0.001) in hypoxia group; however, the DCF intensity was still higher than that in the control group. These data suggested that H_2_S inhibits hypoxia-induced ROS.

### 3.3. The Role of ROS on [Ca^2+^]_i_ Induced by Hypoxia

In order to explore the effect of hypoxia on [Ca^2+^]_i_ in 16HBE14o- cells, calcium ion fluorescence probe Fura-2/AM and fluorescent microscopy imaging system (CellR-MT20) were used to detect [Ca^2+^]_i_. 16HBE14o- cells were treated with 1 mM NAC (a specific ROS scavenger) and 400* μ*M CoCl_2_ individually or simultaneously. The F340/380 ratio was used to indicate the concentration of cytosolic calcium.

**Figure 3:**
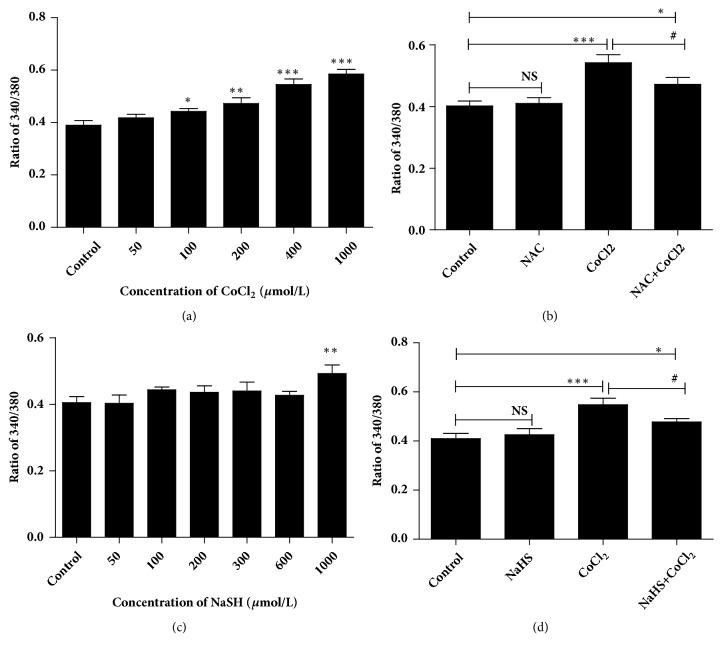
Hydrogen sulfide inhibited ROS-induced intracellular [Ca^2+^]_i_ in 16HBE14o- cells. Logarithmic growth cells were incubated with calcium ion fluorescence probe Fura-2/AM and the F340/380 ratio value was detected by laser scanning confocal microscope. (a) The effect of CoCl_2_ on intracellular [Ca^2+^]_i_ in different doses (0, 50, 100, 200, 400, or 1000* μ*mol/L). (b) NAC (1 mM, scavenger of ROS) inhibited the increase in F340/380 ratio induced by hypoxia (CoCl_2_, 400* μ*mol/L). (c) Effects of hydrogen sulfide on [Ca^2+^]_i_ in different doses (0, 50, 100, 200, 300, 600, or 1000* μ*mol/L). (d) Effects of hydrogen sulfide (NaHS, 300* μ*mol/L) on [Ca^2+^]_i_ induced by hypoxia (CoCl_2_, 400* μ*mol/L). The results were from 3 independent experiments (NS, *∗p* < 0.05, *∗∗p* < 0.01,*∗∗∗p* < 0.001 versus control group, #*p* < 0.05 versus hypoxia group, n=3).

Hypoxia promoted F340/380 ratio value in a dose-dependent manner (Figure 3(a)). The [Ca^2+^]_i_ was decreased from 0.54 ± 0.058 (hypoxia group) to 0.47 ± 0.049 by treatment of NAC (Figure 3(b)). Compared with the control group, F340/380 ratio value in the NAC group has no significant difference. These data indicated that hypoxia increased the [Ca^2+^]_i_ mediated by its intracellular ROS.

### 3.4. H_2_S Attenuates [Ca^2+^]_i_ Induced by Hypoxia in 16HBE14o- Cells

To determine the effect of H_2_S on [Ca^2+^]_i_ during hypoxia, we firstly treated the 16HBE14o- cells with NaHS in different concentrations. As shown in Figure 3(c), a trifling elevation in [Ca^2+^]_i_ was detected in 16HBE14o- cells except for at the concentration of 1000* μ*M. Similar to NAC, H_2_S could effectively suppress CoCl_2_-induced increase in F340/380 ratio value (from 0.55± 0.059 to 0.48± 0.031), and the [Ca^2+^]_i_ was still higher than that in the control group (Figure 3(d)). However, treatment with NaHS (300* μ*M) alone had no significant effect on [Ca^2+^]_i_. The above-mentioned results (Figure 3) demonstrate that H_2_S attenuates [Ca^2+^]_i_ induced by hypoxia via reducing ROS.

### 3.5. H_2_S Promoted MMP of 16HBE14o- Cells Inhibited by Hypoxia

**Figure 4:**
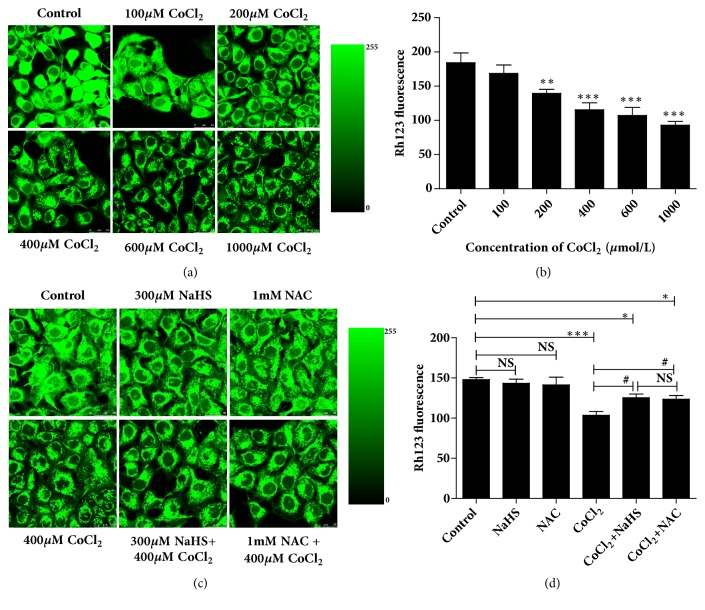
Effects of hydrogen sulfide on MMP induced by hypoxia in 16HBE14o- cells. MMP in 16HBE14o- cells was detected by incubating the cells with Rh123 and analyzed with the fluorescent microscopy software. (a, b) Cells were pretreated with CoCl_2_ in different concentration (0, 100, 200, 400, 600, or 1000* μ*mol/L). (c, d) 16HBE14o- cells were pretreated with NaHS (300* μ*mol/L), NAC (1mM), and CoCl_2_ (400* μ*mol/L) separately or together. Experiments were repeated for three times (NS, *∗p* < 0.05, *∗∗p* < 0.01, *∗∗∗p* < 0.001 versus control group, #*p* < 0.05 versus hypoxia group, n=3).

We used the fluorescent microscopy software and fluorescence dye Rh123 to investigate the MMP in 16HBE14o- cells during hypoxia. We found that cells with hypoxia decreased the fluorescence intensity of Rh123 in a concentration-dependent manner (Figures 4(a) and 4(b)). The fluorescence intensity decreased by 8.49%, 24.28%, 37.30%, 41.85%, and 49.48% versus control group corresponding with the CoCl_2_ concentration of 100, 200, 400, 600, and 1000* μ*M (Figures 4(a) and 4(b)).

Data of Figures 4(c) and 4(d) mean that NaHS (300* μ*M) and NAC (1 mM) markedly inhibited the decrease of MMP in 16HBE14o- cells during hypoxia (CoCl_2_, 400* μ*M), while the MMP was still higher than that in the control. In addition, there were no effects with the pretreatment of NAC or NaHS alone. These results indicate that the MMP in 16HBE14o- cells decreases during hypoxia, which was arrogated by H_2_S through reducing the ROS content.

### 3.6. Effects of H_2_S on Cell Apoptosis Induced by Hypoxia

**Figure 5:**
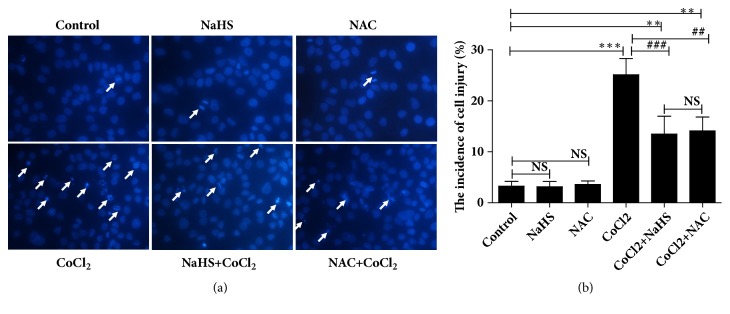
The effects of hydrogen sulfide on hypoxia-induced apoptosis. Exponential growth cells were stained with Hoechst 33342. The nuclei in control, NAC (1 mM), and NaHS (300* μ*M) groups have the normal phenotype demonstrating bright colors and homogeneity. The apoptotic nuclei emitted bright fluorescence and condensed. The number of apoptotic nuclei was significantly increased by CoCl_2_ (400* μ*M) treatment. NAC and NaHS significantly decreased the apoptosis of 16HBE14o- cells (NS, *∗∗p* < 0.01, *∗∗∗p* < 0.001 versus control group, ##*p* < 0.01, ###*p* < 0.001 versus hypoxia group, n=3).

To confirm the role of H_2_S in hypoxia-induced apoptosis, 16HBE14o- cells were incubated with Hoechst 33342 and treated with NAC (1 mM) or NaHS (300* μ*M) with/without hypoxia (CoCl_2_, 400* μ*M). As shown in Figure 5, cells in control had normal phenotype and showed diffused staining of the chromatin. Hypoxia significantly elevated the ratio of damaged cells, but NAC and NaHS could effectively inhibit hypoxia-induced cell injury. After exposure to hypoxia, more cells presented typical morphological changes of cell injury such as chromatin condensation, cell shrinkage, chromatin margination, or apoptotic bodies. However, the cells of NAC or NaHS group had no more serious apoptosis than that in the control group. This suggested that H_2_S alleviate the hypoxia-induced apoptosis by reducing the level of ROS in the cultured 16HBE14o- cells.

## 4. Discussion

In the present study, we explored the contribution of H_2_S in cell injury induced by hypoxia in 16HBE14o- cells. It was demonstrated that in 16HBE14o- cells pretreatment with NaHS during hypoxia (i) the level of ROS decreased, (ii) the [Ca^2+^]_i_ was reduced and MMP was elevated, and (iii) the cell apoptosis was relieved. Our results suggested that the H_2_S plays a protective role in CoCl_2_-induced cell injury in 16HBE14o- cells by reducing the ROS content to regulate the level of [Ca^2+^]_i_ and MMP.

Oxidative stress induced by hypoxia/ischemia resulted from the imbalance between ROS and the antioxidant defense system. Accumulating evidence has suggested that ROS induced by hypoxia/ischemia stroke is closely associated with the exacerbation of atherosclerosis, cardiovascular disease [31], and the pathogenesis of airway disorders such as adult respiratory distress syndrome (ARDS), cystic fibrosis, idiopathic fibrosis, COPD, and asthma [1, 32–34]. Airway cells and tissues were exposed to oxidative stress such as environmental pollutants, infections, inflammatory reactions, or decreased levels of antioxidants and the excessive ROS could cause a variety of deletion effects in the airway [12]. In order to mimic hypoxia, we treated the 16HBE14o- cells with CoCl_2_ for a short period of time which is a common chemical mimic of hypoxia* in vitro* [28, 35]. We found that CoCl_2_ had the significant dose-dependent inhibitory effect and H_2_S had the protective effect on cell viability in 16HBE14o- cells. Our data showed that hypoxia significantly elevated the level of ROS, leading to intracellular Ca^2+^ accumulation and MMP decrease in cultured 16HBE14o- cells, and aggravating apoptosis of 16HBE14o- cells.

Accumulating evidence showed that oxidative stress could lead to the MMP disruption and apoptosis [10, 11, 15, 16, 36] and these were attenuated by H_2_S. Robert F and Isabel CP reported that H_**2**_S could induce the airway smooth muscle relaxation and inhibited the Ca^2+^ release in smooth muscle cells [37, 38]. The endogenous production of H_2_S was decreased in the lung tissue of hypoxic pulmonary hypertension (HPH) followed by oxidative stress [20]. Furthermore, injury and apoptosis of epithelial cells and their defective repair are closely related to the pathogenetic process and development of COPD and asthma [39, 40]. It has been demonstrated that H_2_S can react with ROS and work as a scavenger of oxygen-derived free radicals [23, 41, 42]. Our data show that H_2_S exerted inhibitory effects similar to the ROS scavenger NAC on hypoxia-induced ROS elevation and ROS-mediated cytosolic calcium influx and the disruption of MMP. Our studies also found that H_**2**_S remarkably attenuated apoptosis in cultured 16HBE14o- cells induced by hypoxia. These data indicate that cytosolic calcium influx and the disruption of MMP mediated by ROS are involved in hypoxia-induced bronchial epithelial cells apoptosis. H_2_S played the protective role in the process of oxidative stress which may be associated with NF-*κ*B pathway, BDNF pathway, and ROS-activated ERK1/2 pathway [43–45]. However, 2–4 mM of NaHS* in vitro* caused shrinkage and death of the cells [46]. Therefore, the concentration of H_2_S is also crucial. Therefore, the corresponding signaling pathway and the concentration of H_2_S need further study.

In conclusion, our findings confirmed that H_2_S attenuated hypoxia-induced cell injury in 16HBE14o- cells. Moreover, H_2_S antagonized hypoxia-induced accumulation of [Ca^2+^]_i_ and dissipation of MMP to relieve cell apoptosis by increasing the scavenging of ROS. These data reveal that the clearance of ROS is responsible for the protective effect of H_2_S against hypoxia-mediated cell injury.

## Data Availability

The data used to support the findings of this study are included within the article.
